# Implementing bubble continuous positive airway pressure in a lower middle-income country: a Nigerian experience

**DOI:** 10.11604/pamj.2020.37.10.24911

**Published:** 2020-09-03

**Authors:** Chiamaka Aneji, Tyler Hartman, Oluyinka Olutunde, Ikechukwu Okonkwo, Eghosa Ewumwen, Oniyire Adetiloye, Joseph de Graft-Johnson, Chinyere Ezeaka, Angela Okolo, Ngozi Ibeziako, Obumneme Ezeanosike, Patricia Medupin, George Little

**Affiliations:** 1 McGovern Medical School, University of Texas Health Science Center at Houston, Texas, United State of America,; 2 Geisel School of Medicine at Dartmouth Lebanon NH, United State of America,; 3 Maternal and Child Survival Program, Abuja, Nigeria,; 4 University of Benin Teaching Hospital Benin City, Edo State, Nigeria,; 5 Mile 4 Mission Hospital, Abakiliki, Nigeria,; 6 Maternal and Child Survival Program Project Director, JHPIEGO, Nigeria,; 7 Save the Children, Washington DC, United State of America,; 8 College of Medicine, University of Lagos, Lagos, Nigeria,; 9 Federal Medical Centre, Asaba, Nigeria,; 10 University of Nigeria Teaching Hospital, Enugu, Nigeria,; 11 Federal Teaching Hospital, Abakaliki, Nigeria,; 12 Federal Medical Centre, Lokoja, Nigeria

**Keywords:** Bubble continuous positive airway pressure, neonate, lower-middle-income country, program development, program assessment

## Abstract

Bubble CPAP (bCPAP) is used for respiratory distress (RD) in neonates. The leading causes of neonatal mortality can lead to severe RD. Many neonatal deaths are preventable using evidence-based interventions like bCPAP as part of a comprehensive approach. The study aimed to assess the implementation of a multi-center, comprehensive hospital-based bCPAP program in a low-middle-income country using a low-cost bCPAP device. Seven established hospitals in three Nigerian States were selected using purposive sampling. A respiratory support program was developed and implemented using the Pumani® bCPAP. Neonates <28 days old with severe RD, birth weight >1000g and breathing spontaneously, were eligible. The program lasted 22 months. Focus group discussions and in-depth interviews of healthcare workers and hospital administrators were used in program assessment. Content analysis of qualitative data completed. The staff reported that the bCPAP device was easy to use and effective. All staff reported comfort in eligible patient identification, effective set up and bCPAP administration. All study sites experienced varying degrees of electric power interruption and oxygen availability and affordability. Staff training, staffing disruptions, data collection challenges and use of improvised bCPAP contributed to low enrollment. Advocacy, direct program support, and innovation using locally available resources improved enrollment. Professional organization collaboration, competency-based training and peer mentoring contributed to program success. Thorough pre-program assessment, with comprehensive understanding of all aspects of the existing system within the local context which are likely to impact the introduction of a new program is important to implementation success.

## Project evaluation

### Introduction

There are over a quarter million annual neonatal deaths in Nigeria, about 10% of the global occurrence. The leading causes of neonatal mortality in Nigeria are intrapartum-related events (32%), prematurity (31%), sepsis (16%) and tetanus (3%) (2018) [1]. Three-quarters of global newborn deaths are due to preventable causes, manageable by proven cost-effective interventions [2]. Respiratory distress (RD) is common to all major causes of mortality, and often responds to effective management [3]. Neonatal RD in Nigeria contributes to the high burden of neonatal morbidity and mortality. Continuous positive airway pressure (CPAP) was first described as effective in 1971 [4]. CPAP is considered the initial mode of respiratory support for RD in preterm infants in most high-income settings and is increasingly being used in low- and-middle-income countries (LMICs) [3]. A 2002 Cochrane review showed that CPAP in spontaneously breathing preterm infants with respiratory distress syndrome reduced mortality by 48% [5]. Bubble CPAP (bCPAP) is one of several methods of CPAP generation that has been in use worldwide for more than 30 years [6]. It is safer than mechanical ventilation [7], simple to operate [8] and the commercial model is about one- tenth the cost of mechanical ventilators [9]. These factors make bCPAP an excellent candidate for use in LMICs [6].

LMIC studies evaluating bCPAP in neonates < 28 days age with RD concluded bCPAP is safe and can reduce need for mechanical ventilation [3,10,11]. However, more studies assessing effective implementation strategies in LMIC settings are needed [11]. The Nigerian health care system strives to refer sick newborns to secondary- and tertiary-level hospitals while also focusing on community-based care. A survey of health care providers in tertiary hospitals reported that supplemental oxygen via nasal cannula or nasopharyngeal tube was the most common respiratory support option [12]. Non-commercial, locally improvised bCPAP (ibCPAP) is the most commonly used bCPAP in Nigeria. These are simple, low-cost devices assembled from locally available components powered by high flow of 100% oxygen up to 5-7 liters per minute (lpm) from a cylinder or oxygen concentrator [12-17]. However, these have safety design limitations including lack of oxygen blending in some cases with risk of oxygen toxicity, and lack of air pressure regulation, warming and humidification, which can cause tissue damage [18,19]. Commercially available models are in use but their cost severely limits widespread availability [12,20]. To improve care for neonates in RD within the context of a comprehensive maternal and newborn project, a multisite bCPAP intervention in three States in Nigeria was designed and implemented. This report details the process, benefits, and challenges encountered during implementation, and describes lessons learned with recommendations.

**Bubble CPAP (bCPAP) implementation process**: the bCPAP implementation was conducted in cooperation with the Nigerian Federal and State Ministries of Health, and the United States Agency for International Development-funded Maternal and Child Survival Program (MCSP). bCPAP was introduced as part of a newborn health package in seven hospitals in three States (Table 1). MCSP partnered with the Pediatric Association of Nigeria (PAN), the Nigerian Society of Neonatal Medicine (NISONM) and the American Academy of Pediatrics (AAP) to adapt protocols, training materials and job aids/tools from a similar project in Malawi [21]. Hospital selection was by purposive sampling. The main criterion was hospital delivery volume. No site had commercial bCPAP (Table 1). Neonates <28 days old with severe RD, birth weight >1000g, breathing spontaneously and without obvious neurological complications were eligible. Exclusion criteria included presence of specific congenital anomalies, severe cardiac instability, and severe asphyxia. This was a standardized program for initiation of a medical standard of care, so parental consent was not sought. The Pumani bCPAP® device (designed 2015-2017) was chosen because of its reported reliability, low maintenance requirements and low cost ($800 in 2016) [21]. A non-randomized trial using the Pumani® in Malawi reported an absolute risk of death reduction of 30%, and limited complications like nasal irritation and epistaxis [21]. Protocols and checklists to manage moderate to severe neonatal RD were developed and data collection tools designed. One doctor and two nurses per hospital were trained as site champions. They completed a two-day hands-on intensive training workshop off-site. Eligible patient identification and management, device trouble shooting and maintenance, and data acquisition were emphasized. Champions were tasked with replicating hands-on training at their home hospital, provide peer-mentoring/training and act as liaisons with MCSP staff. Site champions then trained their site (Neonatal Intensive Care Unit) NICU and special care baby unit (SCBU) staff at six of the seven sites, and MCSP partnered with NISONM staff to train providers at the seventh site. NICU staff were responsible for patient enrollment, management and data collection. From August 2016 through June 2018, MCSP field staff physicians provided program organizational support and implementation guidance. Support provided included data retrieval, reviewing training status, monitoring protocols and equipment use/maintenance and tracking clinical outcomes especially adverse events. The program underwent comprehensive implementation assessments after one year and at implementation completion. A structured, qualitative questionnaire focused on provider competency in patient identification, initiation and management of bCPAP, device acceptance and perceived effectiveness, program cost, administrative leadership and facility related challenges was used. A neonatologist with extensive experience with the Nigerian health care system conducted all interviews. Site visits lasted approximately three hours/site. Content analysis of qualitative data was completed.

**Table 1:** selected hospital description

State	Name (Hospital type)	Level of care	Baseline ibCPAP usage	Number Pumani devices
**Ebonyi**	Federal Teaching Hospital Abakaliki (Federal)	Tertiary (Neonatal intensive care unit (NICU))	Yes	2
	Mile Four Mission Hospital (Mission)	Secondary	No	2
	Mater Misericordiae Hospital (Mission)	Secondary	No	1
**Kogi**	Federal Medical Center (Federal)	Tertiary (NICU)	Yes	2
	Evangelical Church Winning All (ECWA) Hospital (Mission)	Secondary	No	1
**Cross River**	University of Calabar Teaching Hospital (Federal)	Tertiary (NICU)	Yes	2
	General Hospital Calabar	Secondary	No	1

### Implementation benefits

**Medical staff development**: the program´s hands-on training led to staff development at all sites in the use of bCPAP and patient care/monitoring. Most NICU doctors and nurses had no prior experience using a patented bCPAP device and had not received rigorous hands-on training on patient identification, monitoring, and management on such devices. Trained staff all self-reported high level of comfort/competence diagnosing RD and using bCPAP therapy. In our survey, nurses reported comfort operating the bCPAP device.

**Improvement in quality and safety patient care**: providers´ comments included “algorithms and checklists had clear escalation and weaning protocols and improved patient care consistency,” “the patient monitoring tool improved the quality of patient care,” ”It is clear to see trends in patient vital signs and it made handoffs a lot easier and faster,” and “it made care more efficient.” Other providers commented “It is so easy and quick to set up the machine compared to the ibCPAP,” and “you see rapid improvement once patient is on the machine.” No hospital had oxygen-blending capability prior to the program. The in-built oxygen blender in the Pumani made oxygen titration possible, thus limiting exposure to harmful oxygen concentrations associated with high-flow NC or ibCPAP without blenders.

**Hospital capacity building**: significant and meaningful expansion of evidence-based therapeutic options for RD occurred at all sites. One administrator stated, “this technology has really improved the scope of services we provide.” MCSP provided electric-powered oxygen concentrators to all sites, improving oxygen availability. While program funding ensured access to bCPAP therapy for all eligible patients, electricity and oxygen availability/cost were factors limiting universal accessibility (see below). The program assured supportive care for all identified eligible patients. Some improvement occurred in availability of electricity with MCSP advocacy with hospital administrators to repair or purchase alternate sources of energy.

**Bubble CPAP (bCPAP) acceptance**: there was a strong positive acceptance of bCPAP technology among NICU staff at all sites. Staff commented, “It is much easier and quicker to assemble,” “It is more effective than the improvised CPAP.” Staff reported patient families had favorable attitudes, although some initially expressed concern and suspicion about a perceived new technology.

### Implementation challenges

**Limited electricity**: there were major challenges to implementation and patient enrollment. Electricity issues severely limited patient enrollment at all sites because the Pumani device requires electricity. All hospitals had a petrol-powered generator but only six of seven had electricity from the national grid. However, high petrol costs limited the number of hours that generators operated at all sites. Fifty percent of sites had batteries/inverters. The most severely affected site had electricity <6 hours/day from a petrol-powered generator. No site had reliable 24 hour/7 day/week supply. Only one hospital had nearly 24-hour availability of power prior to program implementation but experienced significant disruption due to uncoordinated NICU renovation resulting in > 50% decline in electricity availability and stability. The degree of power problems was under-appreciated during program planning. Consistent electricity was considered in hospital selection and a contingency plan to use low flow NC as back up with outages were part of program protocol. However, some providers, when confronted with erratic outages or fluctuating supply, transitioned patients off the Pumani to high flow NC or ibCPAP, and did not switch back to Pumani when power returned. Others with erratic power would not start patients on Pumani at all. Providers´ rationale was, “we knew the electricity would be back only briefly.” To improve the situation, hospital administrators were urged to look at alternate sustainable power sources. One hospital invested in solar-powered batteries. Others invested in inverters. However, absence of automated power switch-over mechanisms and uninterrupted-power-supply devices at all sites caused discontinuity in care.

**Limited availability of oxygen**: oxygen availability clearly interfered with implementation. Piped oxygen is not readily available in many hospitals in Nigeria. Most rely on oxygen supplied in cylinders or an oxygen concentrator. No implementation site had piped oxygen, though one was in the process of installation. Only one facility had on-site oxygen generation capability. Problems with oxygen cylinder supply-chain included limited number of cylinders at some facilities. Many sites had only one oxygen concentrator. Oxygen is relatively expensive nationally, pricing is typically done per/cylinder or per/diem and families typically pay out-of-pocket. Three of seven sites required payment well beyond the means of most families. Hospital administrations often struggled to subsidize oxygen costs and improve availability and affordability. Advocacy, direct support from MCSP, and financing through hospital administration helped subsidize oxygen costs. Improved oxygen cylinder supply-chains and portable electric oxygen concentrator installation both improved availability and affordability at all sites.

**NICU staff training**: the quality of initial site trainings by site champions varied. Severe time constraints (class duration one to two hours only), large class sizes, limited teaching aids like audio-visual resources, limited numbers of trainers (conducted solely by the physician champion, not nurse champions/site), and limited numbers of bCPAP devices (one to two per site) led to inadequate staff practice. Staff assessed the training as inadequate at three of six sites that held these trainings. Staff comments included; “We were too many in a class with only one trainer,” and, “There was no time for hands-on practice.” The lack of standardization resulted in low patient enrollment and in many staff not using bCPAP according to program protocol. Following the midpoint program assessment and recommendations, MCSP staff partnered with site champions to organize refresher hands-on workshops using multiple trainers and equipment, lasting one day/site. Comments following the workshops included, “MCSP staff support for the retraining workshop made a great difference,” “we learned a lot,” and “practicing with the machine was very good.”

**Patient enrollment**: enrollment was lower than expected across all program sites. Seventy-six neonates, (31% of the projected sample size) were enrolled. Nationwide hospital worker strikes with resulting severe service limitations and inadequate staff availability, training and commitment were also contributing factors. Site-specific staffing issues also contributed. Prior to the refresher training, staff did not feel confident enough to enroll patients. Another site referred all eligible patients to a referral hospital, resulting in temporary site closure. This site reopened following the refresher training. Recruitment at one site halted due to a device malfunction that took months to repair due to communication and distance challenges.

**Data collection**: data collection was a persistent challenge despite MCSP´s efforts. Several factors were cited, including complete reliance on already limited number of NICU staff to manually collect all program data. The original patient monitoring and outcome data tools were complex and not user-friendly at seven pages. The tools were revised by MCSP staff into an efficient one-page format with simplified data fields. However, there was shortage of data collection forms at some sites. Retrieval of completed tools after patient discharge or death was difficult. This was due to hospital policy that all patient documentation including bCPAP-monitoring sheets remain in patients´ hospital folders and stored securely at the hospital. A quality improvement (QI) process or a data use agreement put into place with program initiation could have addressed this.

**Human resources**: NICU staff retention was a common complicating factor due to trained staff rotation to different services at some sites. Most sites had no mechanism for training or mentoring new staff on bCPAP use.

**Equipment maintenance and repair**: Nigeria, like many LMICs, is challenged by limited availability of repair services for medical equipment. Program champions were trained to identify and repair minor equipment malfunctions. Two of 11 program devices malfunctioned, requiring repair beyond the scope of the champions´ training. MCSP staff facilitated these repairs by taking the malfunctioning device to a vendor for repair. A remote site went one year with no functioning device due to repair scheduling challenges arising from communication and distance difficulties. Lost nasal prongs or prongs too big for the smallest eligible patients, insufficient hats, and incompatible connection tubing to oxygen cylinders also were issues. Staff at several sites innovated to work around the hats, prongs and tubing problems by adapting/improvising with locally available materials. This supports the value of active scenarios or reality-based training that employs actual equipment in future implementation efforts.

**Social media for group communication**: attempts to facilitate electronic group communication utilizing WhatsApp® among champions at the beginning of bCPAP implementation was unsuccessful. This was because of under-utilization by staff at all sites. To our knowledge, only one site champion used the platform for program communication.

**Financial Considerations**: planned data collection on patient-care treatment costs was not done at six of seven sites. The reason cited included severe time constraints and no dedicated project staff for cost analysis. Therapy cost information would be important for program implementation to help health leaders in sustainably funding programs.

### Discussion

In implementing bCPAP for various care settings and patient populations in Nigeria, we learned valuable lessons. These are applicable for not only Nigeria but also for LMICs with similar circumstances. The implementation experience revealed a number of influences that were systemic in origin, such as acceptance of an apparatus, ibCPAP, pervasive problems with electricity and oxygen availability, and a problem of staff availability. Could these systemic influences have been better identified and accounted for prior to implementation?

Selected recommendations to address challenges and strengthen bCPAP implementation at a facility level include: 1) Careful anticipatory assessment of the system of healthcare within which the implementation is to occur is crucial. The Nigerian effort focused on multiple facility-based implementation rather than a systematic implementation and change. 2) Dedicated power supply including a voltage stabilizer and uninterrupted power with affordable alternative reserve and renewable electricity sources is crucial for continuous availability of interventions requiring electricity. 3) Reliable and affordable oxygen supply including systems for ensuring uninterrupted supply to NICUs is also crucial for continuous respiratory support. Stakeholders making this a national health care priority may lead to lower costs and more widespread availability. Oxygen concentrators, which have been described as best option in LMICs [22], should be part of the solution. 4) Evidence-based bCPAP protocols and guidelines tailored to the local need to ensure quality and consistency of care are important. This should include patient identification algorithms, monitoring, support escalation and de-escalation. Periodic evaluation and improvement using QI principles could increase adherence and accelerate progress. 5) Combination of advocacy, collaboration with hospital management and direct program support of any implementation is key. 6) Available, accessible, affordable and effective device repair and maintenance services are essential for sustainable implementation. 7) Effective communication and messaging for staff and caregivers with visual aids and scripting to improve ready acceptance of new technology is important. 8) Professional association involvement is important and should be part of any implementation process. 9) Peer mentoring with refresher trainings is critical to maintaining ongoing staff education. 10) Future efforts should include a QI process that supports providers to use data collected to monitor the performance and make onsite care adjustments to ensure neonates receive quality bCPAP care. 11) Using social-media platform for support and education and having a key champion for programs may improve future success.

This program assessment has identified challenges that must be addressed to ensure future successful and sustained implementation of bCPAP in resource-limited settings. A bCPAP device that combines the ability to titrate oxygen without reliance on electricity would be groundbreaking. This would be ideal for environments like Nigeria where the cost of electricity from alternative sources is high and the local grid electricity supply often unreliable. Until such a device is available and rigorously tested, renewable sources of electricity should be strongly considered as a viable supplement in local situations. Some of the findings of the Nigerian program are similar to reports from other LMICs. South Indian and Fiji nurses in two separate studies reported nurses, with training, were able to set up bCPAP [23,24]. Providers believed bCPAP was effective. Nurses endorsed they could care for and wean patients without doctor´s present. Staff retention challenges and lack of awareness and knowledge about CPAP barriers were reported [24]. A strength of the program was the deliberate involvement of PAN and NISONM professional associations´ staff and members in the initiative. For example, they strongly emphasized oxygen as essential therapy for RD. Their input contributed significantly to the initiation of bCPAP and the adjustments made during implementation to improve the care provided to neonates in need of breathing assistance because of RD. Members of these organizations can then incorporate solutions that worked into policies and guidelines for wider dissemination.

### Conclusion

Improving care for neonates with RD using bCPAP devices is challenging in low-resource settings [6]. It is essential to consider all systems issues before initiating the introduction of a new life-saving device. Health leaders and managers should take the challenges outlined by this program experience into account when planning bCPAP or other health technology implementation. bCPAP in LMICs has a lot of potential. Areas for further implementation research could include: education models to ensure skills and knowledge retention, innovative staffing models for resource-limited settings, and sustainable solutions for widespread infrastructure problems such as oxygen and electricity challenges. **Funding**: this manuscript is made possible by the generous support of the American people through the United States Agency for International Development (USAID) under the terms of the Cooperative Agreement AID-OAA-A-14-00028. The contents are the responsibility of the authors and do not necessarily reflect the views of USAID or the United States Government.
